# MULTI-CHANNEL MARKETING EXPOSURE AND PSYCHOACTIVE SUBSTANCE USE IN E-CIGARETTES: A CROSS-SECTIONAL STUDY OF POLISH ADOLESCENTS AND YOUNG ADULTS

**DOI:** 10.13075/ijomeh.1896.02532

**Published:** 2025

**Authors:** Karolina Zajdel, Anna Merecz-Sadowska, Arkadiusz Sadowski, Dorota Kaleta

**Affiliations:** 1 Medical University of Lodz, Department of Medical Informatics and Statistics, Łódź, Poland; 2 University of Lodz, Department of Economic and Medical Informatics, Łódź, Poland; 3 Medical University of Lodz, Department of Allergology and Respiratory Rehabilitation, Łódź, Poland; 4 Medical University of Lodz, Department of Hygiene and Epidemiology, Łódź, Poland

**Keywords:** teenagers, psychoactive substances, adolescents, public health policy, e-cigarettes, marketing exposure

## Abstract

**Objectives:**

The increasing prevalence of electronic cigarettes (e-cigarette) use among adolescents has raised concerns about potential high-risk behaviors, particularly the addition of psychoactive substances to e-cigarette liquid (e-liquids). This study examines the association between exposure to e-cigarette marketing and the practice of adding psychoactive substances to e-liquids among Polish teenagers.

**Material and Methods:**

A cross-sectional survey was conducted with 8344 Polish teenagers aged ≥15 years. The study evaluated exposure to various e-cigarette marketing channels, the prevalence of psychoactive substances added to e-liquids and associated demographic and socioeconomic factors.

**Results:**

Among participants, 7.15% reported adding psychoactive substances to e-liquids, with tetrahydrocannabinol being the most commonly reported substance. E-cigarette marketing exposure, especially in urban areas and via digital platforms, was significantly associated with an increased likelihood of adding psychoactive substances to e-liquids (p < 0.001). Higher-income adolescents exhibited greater susceptibility to this behavior when exposed to marketing. Maternal secondary education demonstrated a protective effect. Various marketing channels showed differential impacts, with club or social venue promotions demonstrating the strongest association with psychoactive substance use.

**Conclusions:**

These findings elucidate the complex interplay between e-cigarette marketing exposure, socioeconomic factors, and high-risk e-cigarette use among adolescents. The results underscore the necessity for more stringent regulation of e-cigarette marketing and comprehensive, targeted prevention strategies focusing on urban and higher-income youth populations.

## Highlights

- Up to 7.15% of Polish teens add psychoactive substances to electronic cigarettes (e-cigarette) liquids (e-liquids).
- Urban areas show highest rates of e-liquids substance modification.
- Higher income correlates with greater risk of e-liquid substance addition.
- Marketing exposure linked to increased psychoactive substance use.

## INTRODUCTION

Electronic cigarettes (e-cigarettes), introduced in the mid-2000s, have become a significant public health concern. These devices vaporize a liquid solution containing nicotine, propylene glycol, vegetable glycerin, and flavorings [1]. Initially marketed as smoking cessation aids, e-cigarettes have gained substantial popularity among adolescents and young adults [1,2].

The prevalence of e-cigarette use has increased dramatically the past few years. In the USA, current e-cigarette use among high school students rose from 1.5% in 2011 to 19.6% in 2020 [2,3]. Similar concerning trends have emerged across Europe [4]. The 2021/2022 Health Behavior in School-aged Children (HBSC) study [5] further confirms this pattern, reporting that approx. 20% of 15-year-olds across Europe used e-cigarettes within the past 30 days, while also demonstrating diminishing gender differences in substance use behaviors among adolescents.

In Poland, data from the 2016 Global Youth Tobacco Survey (GYTS) [6] indicate that 26.9% of students aged 11–17 years reported current e-cigarette use, exceeding the 20.5% who reported conventional cigarette use. The survey identified that 14% of students engaged in dual use of both products. These findings demonstrate a substantial shift in nicotine consumption patterns among Polish youth and indicate potential regression in tobacco control progress. The GYTS data further identify significant sociodemographic factors influencing e-cigarette use, including age, rural residence, and peer or parental smoking habits. The evidence from these national and international studies demonstrates the necessity for targeted interventions and enhanced regulatory measures to address the increasing prevalence of e-cigarette use among youth [6].

This widespread adoption may be attributed to aggressive marketing strategies, perceived reduced harm compared to traditional cigarettes, and appealing flavors [7,8]. The e-cigarette market has evolved substantially, with newer devices offering higher nicotine concentrations and more efficient delivery systems [9]. Regulatory frameworks have struggled to keep pace with these rapid changes [10].

The increasing prevalence of e-cigarettes has prompted extensive research into their addictive potential [11]. While nicotine remains the primary driver of dependence, behavioral and sensory aspects also contribute to addiction [12]. Modern e-cigarette devices, particularly those utilizing nicotine salts, can deliver nicotine with efficiency comparable to conventional cigarettes [13]. Electronic cigarettes users exhibit classic signs of dependence, such as cravings and withdrawal symptoms [14].

Adolescents are particularly susceptible to e-cigarette dependence, developing symptoms even with infrequent use and at lower nicotine exposure levels [15]. Longitudinal studies reveal rapid symptom development, often within months of initiation use [16]. However, the long-term course of e-cigarette dependence remains an active area of investigation [17].

The use of e-cigarettes for delivering psychoactive substances beyond nicotine has emerged as a significant public health concern [18]. Cannabis and its derivatives, particularly tetrahydrocannabinol (THC), are among the most commonly reported substances used in e-cigarette devices. The popularity of cannabis vaping has risen substantially among young people, raising concerns due to increased potency and misconceptions about reduced harm [19]. Synthetic cannabinoids in e-cigarettes are particularly alarming due to their unpredictable and often severe effects [20]. E-cigarette devices are also being used to vaporize other substances such as methamphetamine, cocaine, and synthetic cathinones, posing significant risks of rapid onset of drug effects and increased addiction potential [21].

The discreet nature of e-cigarettes complicates the detection of illicit use, potentially facilitating consumption in public spaces or schools [22]. The combination of multiple substances in e-cigarette liquids (e-liquids) may lead to complex drug interactions and enhanced addictive potential, necessitating further research to understand its prevalence and health implications [23].

This study aims to explore the relationship between multi-channel marketing exposure and psychoactive substance use in e-cigarettes among Polish adolescents and young adults. Specifically, the primary objective is to assess how exposure to various marketing channels, including shop advertisements, club promotions, internet marketing, and influencer marketing, influences the likelihood of adding psychoactive substances to e-liquids. Additionally, the study examines the role of demographic and socio-economic factors in understanding patterns of psychoactive substance use within e-cigarettes.

## MATERIAL AND METHODS

### Study design and participants

This cross-sectional study employed a comprehensive survey to investigate e-cigarette use and associated behaviors among current e-cigarette users (defined as any use within the past 30 days) aged ≥15 years enrolled in secondary educational institutions. A multistage sampling method was utilized to recruit 8344 participants from 200 secondary schools distributed across all 16 voivodships (administrative regions) in Poland, ensuring comprehensive national representation. Schools were selected using stratified random sampling to ensure representation of both urban and rural areas, as well as different types of secondary educational institutions (grammar schools, technical schools, and vocational schools). Among the total sample, 7747 participants reported using only standard e-liquids, while 597 participants reported adding psychoactive substances to their e-liquids. The vast majority of participants used nicotine-containing e-liquids, with only 149 participants in the non-psychoactive substance user group and 7 participants in the psychoactive substance user group using exclusively nicotine-free e-liquids.

### Data collection

This study employed a fully anonymous computer-assisted web interview (CAWI) questionnaire survey, with no personal identifying information collected at any stage. The study was conducted after obtaining administrative approvals from school principals and written informed consent from both parents (for participants <18 years old) and students.

The CAWI technique allowed respondents to independently complete the online version of the questionnaire through an internet link that redirected to prepared questions. The system ensured completion control – respondents could not proceed to the next question without answering the previous one, could not select more answers than indicated, or select mutually exclusive answers. This maintained control over the data collection process despite independent questionnaire completion by respondents. After answering all questions, the data was sent directly to the database in coded form, eliminating the need for manual coding and reducing potential errors.

Data collection was conducted by a research team of 3 members. All team members underwent standardized 1-day training program covering survey administration protocols, ethical considerations, and handling of sensitive information. To ensure consistency and reliability, each team member conducted pilot interviews under supervision before beginning actual data collection. Quality control measures included random spot checks of survey administration by the research coordinator and weekly team meetings to address any concerns or inconsistencies in data collection. Data entry was performed by 2 independent researchers with cross-verification to ensure accuracy. The instrument was pilot-tested for clarity and comprehension on a sample of 150 students before full implementation. Data collection occurred in September 2019 – January 2020.

The overall response rate was 92.4%. Missing data, which occurred in <2% of cases for any given variable, were excluded from the analysis using listwise deletion. The final sample composition closely matched the demographic distribution of Polish secondary school students based on data from the Central Statistical Office of Poland [24].

### Survey instrument

The survey instrument assessed key domains: e-cigarette use patterns, exposure to e-cigarette marketing (shop, internet, club/social venue, and influencer marketing), utilization of psychoactive substances in e-liquids, and demographic information. The questionnaire examined the use of various psychoactive substances, including THC, cocaine, heroin, methamphetamine, mephedrone, fentanyl, amphetamine, codeine, LSD, and benzodiazepines.

### Variables and measurements

Demographic data collected included gender, age group (15–17 years and ≥18 years), school type, place of residence, monthly income, and parental education levels. The age groups were chosen to distinguish between mid-adolescence (15–17 years) and late adolescence/early adulthood (≥18 years). School types were categorized within the Polish educational system as grammar schools, technical schools, and vocational schools. Place of residence was self-reported by participants and categorized into 3 groups: rural areas, cities <500 000 inhabitants, and cities ≥500 000 inhabitants. It should be noted that this reflects participants' permanent residence location, which may differ from their school location, particularly for students who commute from rural areas to urban schools. Monthly income was self-reported and categorized into 2 groups (≤EUR 35 and >EUR 35) based on participants' disposable income. Parental education was classified into 3 levels (primary, secondary, and college) based on the highest completed education level reported by participants.

Current e-cigarette use was defined as any use of e-cigarettes in the past 30 days.

### Marketing and promotion exposure assessment

For the purposes of this study, clear operational definitions of marketing exposure, promotion exposure, and influencer marketing were established to ensure precise measurement and analysis.

Marketing exposure was defined as passive exposure to e-cigarette advertising content, including:

– visual displays in shops such as product displays and posters,
– advertisement presence in clubs and social venues,
– online advertisements including banners and pop-ups,
– influencer content featuring e-cigarettes without direct promotional calls to action.

This type of exposure was characterized by its non-interactive nature and broad reach.

Promotion exposure, in contrast, was defined as active engagement with marketing activities designed to directly influence purchasing behavior. This included:

– in-store promotional activities such as sampling events and direct product demonstrations,
– club promotional events including sponsored parties and product giveaways,
– interactive online promotions such as contests and promotional codes,
– direct promotional content from influencers including discount codes and affiliate links.

These activities were characterized by their interactive nature and direct call to action for potential consumers. Influencer marketing was defined as the strategic use of social media personalities and content creators to promote e-cigarette products and associated lifestyle. This included:

– sponsored content featuring e-cigarettes,
– endorsements by social media influencers,
– narrative-driven product placements in digital content,
– collaborative marketing efforts where influencers share personal experiences or recommendations related to e-cigarette use.

This type of exposure was characterized by its perceived authenticity and potential to create emotional connections with the target audience through trusted digital personalities.

### Statistical analysis

Statistical analyses were conducted using STATISTICA software (v. 13.1, StatSoft Inc., Tulsa, OK, USA). The representativeness of the sample was assessed by comparing the demographic distribution with national statistics for Polish secondary school students. Statistical analyses were conducted both on unweighted data and with post-stratification weights to adjust for minor deviations from population parameters, with no substantial differences found between the approaches. Descriptive statistics were calculated for all variables.

To examine multi-channel marketing exposure patterns, participants were categorized based on their exposure to different combinations of marketing channels (single channel, dual channel, or triple channel exposure). Chi-square tests were used to assess the distribution of exposure patterns between standard e-cigarette users and those reporting psychoactive substance use. *Post hoc* analyses using adjusted standardized residuals were performed to identify specific patterns contributing to significant χ2 results. Residual values exceeding ±1.96 were considered to indicate significant deviations from expected frequencies at p < 0.05, values exceeding ±2.58 at p < 0.01, and values exceeding ±3.29 at p < 0.001.

Logistic regression models were employed to investigate the association between e-cigarette marketing exposure and psychoactive substance use in e-liquids. Odds ratios (ORs) with 95% confidence intervals (CIs) were calculated for all variables. Separate analyses were performed for various types of e-cigarette marketing exposure (shop, club, internet, and influencer marketing), examining their associations with psychoactive substance use while controlling for demographic and socioeconomic factors.

A p-value of <0.05 was considered statistically significant for all analyses.

## RESULTS

### Prevalence and demographic patterns of psychoactive substance use in e-liquids

This study examined the prevalence and demographic patterns (Table 1) of psychoactive substance use in e-liquids among 8344 Polish teenagers who were current e-cigarette users (reported use within past 30 days). Among these current users, 597 (7.15%) reported adding psychoactive substances to their e-liquids. Among those who reported using psychoactive substances (N = 597), THC was the most commonly reported substance, used by 91.96% of users (N = 549), followed by methamphetamine (26.97%, N = 161), mephedrone (25.80%, N = 154), cocaine (23.95%, N = 143), heroin (21.44%, N = 128), and fentanyl (19.60%, N = 117). Other substances, including amphetamine, codeine, LSD, and benzodiazepines, were reported by 5.70% of users (N = 34).

**Table 1.** Prevalence of psychoactive substance use in e-cigarette liquids among Polish teenagers aged ≥15 years – a cross-sectional study in Poland, 2019–2020

Variable	Participants (N = 8344) [n (%)]									
	not users (N = 7747)	users (N = 597)								p
		total	tetrahydrocannabinol (N = 549)	cocaine (N = 143)	heroin (N = 128)	methamphetamine (N = 161)	mephedrone (N = 154)	fentanyl (N = 117)	other (N = 34)	
Gender										<0.0001
female	3540 (45.70)	225 (37.69)	210 (35.18)	39 (6.53)	34 (5.70)	47 (7.87)	45 (7.54)	29 (4.86)	13 (2.18)	
male	4207 (54.30)	372 (62.31)	339 (56.78)	104 (17.42)	94 (15.74)	114 (19.10)	109 (18.26)	88 (14.74)	21 (3.85)	
Age group										0.3406
15–17 years	5718 (73.81)	430 (72.03)	395 (66.16)	115 (19.26)	98 (16.42)	123 (20.60)	120 (20.10)	90 (15.07)	22 (3.69)	
≥18 years	2029 (26.19)	167 (27.97)	154 (25.80)	28 (4.69)	30 (5.03)	38 (6.37)	34 (5.70)	27 (4.52)	12 (2.01)	
School type										<0.0001
grammar	3311 (42.74)	217 (36.35)	202 (33.84)	40 (6.70)	32 (5.36)	49 (8.21)	42 (7.04)	32 (5.36)	10 (1.68)	
other	4436 (57.26)	365 (63.65)	347 (58.12)	103 (17.25)	96 (16.08)	112 (18.76)	112 (36.00)	85 (14.24)	24 (4.02)	
Place of residence										<0.0001
rural	3498 (45.15)	217 (36.35)	199 (33.33)	53 (8.88)	52 (8.71)	53 (8.88)	54 (9.05)	43 (7.20)	10 (1.68)	
city										
<500 000 inhabitants	3997 (51.59)	308 (51.59)	287 (48.07)	58 (9.72)	45 (7.54)	68 (11.39)	59 (9.88)	42 (7.04)	16 (2.68)	
≥500 000 inhabitants	252 (3.26)	72 (12.06)	63 (10.55)	32 (5.36)	33 (5.53)	40 (6.70)	41 (6.87)	32 (5.36)	8 (1.34)	
Monthly income										<0.0001
≤35 EUR	4480 (57.84)	249 (41.71)	222 (37.19)	41 (6.87)	38 (6.37)	47 (7.87)	44 (7.37)	31 (5.19)	10 (1.68)	
>35 EUR	3267 (42.16)	348 (58.29)	327 (54.77)	102 (17.08)	90 (15.07)	114 (19.10)	110 (18.43)	86 (14.41)	24 (4.02)	
Education										
mother										<0.0001
primary	1155 (14.91)	97 (16.25)	89 (14.91)	28 (4.69)	26 (4.36)	29 (4.86)	27 (4.52)	21 (3.52)	7 (1.17)	
secondary	3071 (39.64)	181 (30.32)	166 (27.81)	21 (3.52)	15 (2.51)	29 (4.86)	25 (4.19)	14 (2.34)	9 (1.51)	
college	3521 (45.45)	319 (53.43)	294 (49.25)	94 (15.74)	87 (14.57)	103 (17.25)	102 (17.08)	82 (13.74)	18 (3.02)	
father										<0.0001
primary	1764 (22.77)	142 (23.79)	130 (21.78)	38 (6.37)	36 (6.03)	36 (6.03)	35 (5.86)	27 (4.52)	9 (1.51)	
secondary	3397 (43.85)	201 (33.67)	185 (31.00)	28 (4.69)	23 (3.85)	42 (7.04)	39 (6.53)	22 (3.69)	14 (2.34)	
college	2586 (33.38)	254 (42.54)	234 (39.20)	77 (12.90)	69 (11.56)	83 (13.90)	80 (13.40)	68 (11.39)	11 (1.84)	

Significant gender differences were observed (p < 0.0001), with males consistently demonstrating higher rates of substance use. School type was significantly associated with substance use patterns (p < 0.0001). Parental education levels showed significant associations with substance use (p < 0.0001). Geographical location significantly influenced substance use patterns (p < 0.0001). Large cities (>500 000 inhabitants) exhibited disproportionately high rates of substance use compared to those in rural areas. Income levels were also significantly associated with substance use (p < 0.0001), with higher-income participants showing substantially higher rates of substance use.

These findings reveal significant demographic disparities in psychoactive substance use in e-liquids among Polish adolescents, particularly regarding parental education, geographical location, and income levels. The results emphasize the need for targeted prevention strategies and further research into the underlying factors driving these disparities.

### Multi-channel marketing exposure and psychoactive substance use

Analysis of the interaction and overlap between passive marketing exposure, active promotion exposure, and influencer marketing revealed complex patterns of exposure among e-cigarette users. Although defined as distinct categories, these channels demonstrated substantial interconnection in their reach and impact.

**Figure 1.**
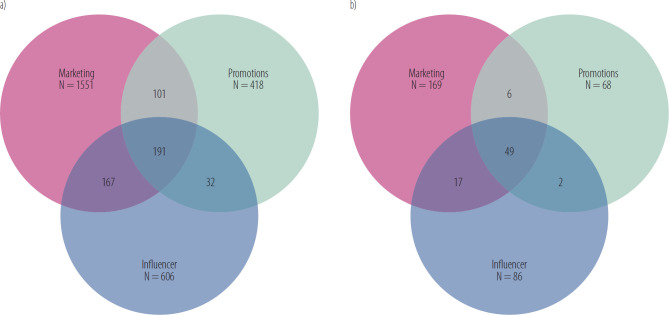
Comparison of marketing channel exposure patterns between a) standard e-cigarette users (N = 7747), b) psychoactive substance users (N = 597) – a cross-sectional study among teenagers aged ≥15 years in Poland, 2019–2020

Examination of multi-channel exposure patterns revealed that among the total sample of 8344 participants, 2093 (25.1%) reported exposure to at least one marketing channel. Of these, 1893 were standard e-cigarette users (24.4% of 7747) and 200 were psychoactive substance users (33.5% of 597), indicating higher overall marketing exposure among substance users. The patterns of exposure to multiple marketing channels were analyzed using Venn diagrams and statistical comparisons between groups (Figure 1).

Numbers in overlapping areas indicate participants exposed to multiple channels simultaneously. Areas are not drawn to scale.

Chi-square analysis revealed significant differences in the distribution of marketing exposure patterns between standard e-cigarette users and those who reported psychoactive substance use (χ^2^ = 38.97, df = 6, p < 0.001). *Post hoc* analyses using adjusted standardized residuals revealed significant deviations from expected frequencies for triple-channel exposure. A strong positive deviation was observed among psychoactive substance users (adjusted standardized residual = 5.44, p < 0.001), indicating a significant overrepresentation in this group. Conversely, a negative deviation, approaching significance, was found among standard users (adjusted standardized residual = –1.77, p < 0.10), suggesting an underrepresentation trend. Deviations for single-channel and dual-channel exposure patterns were minimal and non-significant (p > 0.05), with adjusted standardized residuals of 0.54 and 0.34 for single-channel, and –1.65 and –1.08 for dual-channel exposure, respectively.

Among standard e-cigarette users, 1402 (74.1%) reported single-channel exposure, 300 (15.8%) reported dual-channel exposure, and 191 (10.1%) reported triple-channel exposure. In contrast, among psychoactive substance users, 126 (63.0%) reported single-channel exposure, 25 (12.5%) reported dual-channel exposure, and 49 (24.5%) reported triple-channel exposure.

The proportions of dual-channel exposure remained relatively consistent between groups, with slightly lower rates among psychoactive substance users for combinations of marketing with promotions (3.00% vs. 5.34%) and promotions with influencer marketing (1.00% vs. 1.69%). Marketing combined with influencer exposure showed similar proportions in both groups (8.50% vs. 8.82%).

Statistical analysis demonstrated distinct patterns of marketing exposure between the groups, with particular emphasis on the substantially higher prevalence of triple-channel exposure among psychoactive substance users. These patterns suggest potential cumulative effects of marketing exposure on high-risk e-cigarette use behaviours.

#### Association between marketing exposure and psychoactive substance use

The analysis of e-cigarette marketing exposure and its association with psychoactive substance use among Polish adolescents revealed distinct patterns across various marketing channels (Table 2). Four primary marketing exposure categories were examined: shop advertisements, club or social venue promotions, internet marketing, and other forms of advertising.

**Table 2.** Association between e-cigarette marketing exposure and psychoactive substance use among Polish teenagers aged ≥15 years (N = 8344) – a cross-sectional study in Poland, 2019–2020

Variable	Marketing													
	shop				club				internet				other	
	psychoactive substances use [n (%)]		OR (95% CI)	p	psychoactive substances use [n (%)]		OR (95% CI)	p	psychoactive substances use [n (%)]		OR (95% CI)	p	psychoactive substances use^a^ [n (%)]	
	no	yes			no	yes			no	yes			no	yes
Gender														
male (ref.)	358 (54.91)	53 (62.35)			115 (51.80)	35 (61.40)			598 (54.36)	54 (50.47)			77 (53.10)	14 (82.35)
female	294 (45.09)	32 (37.65)	0.74 (0.46–1.17)	0.1936	107 (42.20)	22 (38.60)	0.68 (0.37–1.22)	0.1946	502 (45.64)	53 (49.53)	1.17 (0.79–1.74)	0.4401	68 (46.90)	3 (17.65)
Age group														
15–17 years (ref.)	442 (67.79)	64 (75.29)			161 (75.52)	41 (71.93)			809 (73.55)	82 (76.64)			109 (75.17)	15 (88.24)
≥18 years	210 (32.21)	21 (24.71)	0.69 (0.41–1.16)	0.1608	61 (27.48)	16 (28.07)	1.03 (0.54–1.97)	0.9288	291 (26.45)	25 (23.36)	0.85 (0.53–1.35)	0.4876	36 (24.83)	2 (11.76)
School type														
grammar (ref.)	268 (41.10)	30 (35.29)			90 (40.54)	21 (36.84)			481 (43.73)	38 (35.51)			72 (49.66)	8 (47.06)
other	384 (58.90)	55 (64.71)	1.28 (0.80–2.05)	0.3046	132 (59.46)	36 (63.16)	1.17 (0.64–2.13)	0.6108	619 (56.27)	69 (64.49)	1.41 (0.93–2.13)	0.1014	73 (50.34)	9 (52.94)
Place of residence														
rural (ref.)	298 (45.71)	41 (48.24)			109 (49.10)	22 (38.6)			505 (45.91)	46 (42.99)			56 (38.62)	8 (47.06)
city														
<500 000 inhabitants	332 (50.92)	30 (35.29)	0.66 (0.40–1.08)	0.0950	102 (45.95)	21 (36.84)	1.02 (0.53–1.97)	0.9527	556 (50.55)	45 (42.06)	0.89 (0.58–1.36)	0.5884	81 (55.86)	6 (35.29)
≥500 000 inhabitants	22 (3.37)	14 (16.47)	4.63 (2.19–9.75)	<0.0001	11 (4.95)	14 (24.56)	6.31 (2.53–15.71)	<0.0001	39 (3.54)	16 (8.41)	4.50 (2.34–8.68)	<0.0001	8 (5.52)	3 (17.65)
Monthly income														
≤35 EUR (ref.)	355 (54.45)	28 (32.94)			102 (45.95)	18 (31.58)			603 (54.82)	43 (40.19)			86 (59.31)	9 (52.94)
>35 EUR	297 (45.55)	57 (67.06)	2.43 (1.51–3.92)	0.0002	120 (54.05)	39 (68.42)	1.84 (0.99–3.45)	0.05067	497 (45.18)	64 (59.81)	1.81 (1.21–2.71)	0.0038	59 (40.69)	8 (47.06)
Education														
mother														
primary (ref.)	84 (12.88)	20 (23.81)			25 (11.26)	10 (17.54)			145 (13.18)	23 (21.50)			20 (13.79)	2 (12.50)
secondary	278 (42.64)	25 (29.76)	0.38 (0.20–0.71)	0.0021	84 (37.84)	13 (22.81)	0.39 (0.15–0.99)	0.0425	444 (40.36)	25 (23.36)	0.35 (0.20–0.64)	0.0004	59 (40.69)	7 (43.75)
college	290 (44.48)	39 (46.43)	0.56 (0.31–1.02)	0.0560	113 (50.90)	34 (59.65)	0.75 (0.33–1.72)	0.4991	511 (46.46)	59 (55.14)	0.73 (0.43–1.22)	2261	66 (45.52)	7 (43.75)
father														
primary (ref.)	138 (21.17)	23 (27.06)			46 (20.72)	9 (15.79)			224 (20.36)	35 (32.71)			34 (23.45)	2 (11.76)
secondary	285 (43.71)	23 (27.06)	0.48 (0.26–0.89)	0.0184	93 (41.89)	13 (22.81)	0.71 (0.28–1.79)	0.4726	473 (43.00)	25 (23.36)	0.34 (0.20–0.58)	<0.0001	47 (32.41)	9 (52.94)
college	229 (35.12)	39 (45.88)	1.02 (0.59–1.78)	0.9394	83 (37.39)	35 (61.4)	2.16 (0.95–4.88)	0.0615	403 (36.64)	47 (43.93)	0.75 (0.47–1.19)	0.2185	64 (44.14)	6 (35.30)

a Due to the small sample size in the subgroups, analysis was not possible.

Among the 8344 participants, 2386 (28.60%) reported exposure to e-cigarette marketing. Among those exposed to marketing, exposure patterns varied by channel. Shop advertisements reached 822 respondents (34.45% of those exposed to marketing), including 737 standard e-cigarette users and 85 who reported psychoactive substance use. Internet marketing demonstrated the highest reach with 1313 respondents (55.03% of those exposed to marketing), including 1206 standard e-cigarette users and 107 who reported psychoactive substance use. Club or social venue promotions reached 337 respondents (14.12% of those exposed to marketing), including 279 standard e-cigarette users and 58 who reported psychoactive substance use, representing the highest proportion of substance users among all marketing channels. Other forms of marketing showed the lowest reach with 180 respondents (7.54% of those exposed to marketing), including 163 standard e-cigarette users and 17 who reported psychoactive substance use.

These findings indicate varying levels of association between different marketing channels and psychoactive substance use in e-cigarettes. While internet marketing reached the largest audience, club or social venue promotions demonstrated the strongest association with psychoactive substance use, followed by shop advertisements. Other forms of marketing showed both the lowest reach and relatively moderate levels of association with psychoactive substance use. This information is crucial for understanding the impact of marketing strategies on high-risk behaviors among adolescent e-cigarette users.

To further examine these relationships, detailed statistical analyses of marketing exposure patterns and their associations with psychoactive substance use across different demographic groups were conducted. Analysis of e-cigarette marketing exposure and psychoactive substance use among Polish adolescents revealed several statistically significant relationships. Exposure to marketing in urban areas with populations ≥500 000 inhabitants was consistently associated with higher rates of psychoactive substance use across all marketing channels. Adolescents in large cities (≥500 000 inhabitants) showed significantly higher odds of psychoactive substance use compared to rural areas across marketing channels: shop marketing (OR = 4.63, 95% CI: 2.19–9.75), club marketing (OR = 6.31, 95% CI: 2.53–15.71), and internet marketing (OR = 4.50, 95% CI: 2.34–8.68), with all associations statistically significant (p < 0.0001).

Socioeconomic factors played a significant role. Adolescents with higher personal disposable income (>35 EUR monthly) exposed to marketing demonstrated higher odds of psychoactive substance use, particularly evident in shop marketing (OR = 2.43, 95% CI: 1.5–3.92, p = 0.0002) and internet marketing (OR = 1.81, 95% CI: 1.21–2.71, p = 0.0038).

Parental secondary education showed a protective effect compared to primary education. This was observed across shop marketing (OR = 0.38, 95% CI: 0.20–0.71, p = 0.0021), club marketing (OR = 0.39, 95% CI: 0.15–0.99, p = 0.0425), and internet marketing (OR = 0.35, 95% CI: 0.20–0.64, p = 0.0004) for maternal education, with similar trends for paternal education.

Gender and age group did not demonstrate statistically significant associations with psychoactive substance use in relation to marketing exposure across most channels. These findings underscore the complex interplay between sociodemographic factors, marketing exposure, and psychoactive substance use in e-cigarettes among Polish adolescents, highlighting urban environments, higher income levels, and parental education as key moderating factors.

#### Association between promotion and psychoactive substance use

The analysis of e-cigarette promotion exposure and its association with psychoactive substance use among Polish adolescents revealed distinct patterns across various promotional channels (Table 3). The study examined 4 primary promotional exposure categories: shop promotions, club or social venue promotions, internet promotions, and other promotional activities.

**Table 3.** Association between e-cigarette promotion exposure and psychoactive substance use among Polish teenagers aged ≥15 years (N = 8344) – a cross-sectional study in Poland, 2019–2020

Variable	Promotion													
	shop				club				internet				other	
	psychoactive substances use [n (%)]		OR (95% CI)	p	psychoactive substances use [n (%)]		OR (95% CI)	p	psychoactive substances use [n (%)]		OR (95% CI)	p	psychoactive substances use^a^ [n (%)]	
	no	yes			no	yes			no	yes			no	yes
Gender														
male (ref.)	80 (68.97)	21 (80.77)			62 (65.96)	26 (72.22)			186 (64.81)	28 (70.00)			41 (64.06)	8 (100.00)
female	36 (31.03)	5 (19.23)	0.53 (0.18–1.51)	0.2300	32 (34.04)	10 (27.78)	0.75 (0.32–1.73)	0.4943	101 (35.19)	12 (30.00)	0.79 (0.38–1.62)	0.5177	23 (35.94)	0 (0.00)
Age group														
15–17 years (ref.)	79 (68.10)	17 (65.38)			72 (76.60)	25 (69.44)			218 (75.96)	27 (67.50)			53 (82.81)	6 (75.00)
≥18 years	37 (31.90)	9 (34.62)	1.13 (0.46–2.77)	0.7889	22 (23.40)	11 (30.56)	1.44 (0.61–3.39)	0.4018	69 (24.04)	13 (32.50)	1.52 (0.74–3.11)	0.2476	11 (17.19)	2 (25.00)
School type														
grammar (ref.)	37 (31.90)	9 (34.62)			35 (37.23)	9 (25.00)			120 (41.81)	10 (25.00)			26 (40.63)	1 (12.50)
other	79 (68.10)	17 (65.38)	0.88 (0.36–2.17)	0.7889	59 (62.77)	27 (75.00)	1.77 (0.75–4.22)	0.1871	167 (58.19)	30 (75.00)	2.16 (1.02–4.58)	0.0418	38 (59.38)	7 (87.50)
Place of residence														
rural (ref.)	42 (36.21)	16 (61.54)			44 (46.81)	14 (38.89)			121 (42.16)	17 (42.50)			29 (40.63)	1 (12.50)
city														
<500 000 inhabitants	68 (58.62)	6 (23.08)	0.23 (0.08–0.64)	0.0029	44 (46.81)	8 (22.22)	0.57 (0.22–1.50)	0.2519	151 (52.61)	12 (30.00)	0.57 (0.26–1.23)	0.1464)	29 (45.31)	1 (12.50)
≥500 000 inhabitants	6 (5.17)	4 (15.38)	1.75 (0.44–7.03)	0.4262	6 (6.38)	14 (38.89)	7.33 (2.37–22.70)	0.0002	15 (5.23)	11 (27.50)	5.22 (2.06–13.22)	0.0002	6 (9.38)	3 (37.50)
Monthly income														
≤35 EUR (ref.)	49 (42.24)	7 (26.92)			38 (40.43)	11 (30.56)			146 (50.87)	11 (27.50)			34 (53.13)	1 (12.50)
>35 EUR	67 (57.76)	19 (73.08)	1.99 (0.77–5.09)	0.0900	56 (59.57)	25 (69.44)	1.54 (0.68–3.50)	0.2988	141 (49.13)	29 (72.50)	2.73 (1.31–5.67)	0.0056	30 (46.88)	7 (87.50)
Education														
mother														
primary (ref.)	15 (12.93)	11 (42.31)			24 (20.69)	11 (30.56)			39 (13.59)	12 (29.27)			9 (14.06)	1 (12.50)
secondary	44 (37.93)	3 (11.54)	0.09 (0.02–0.38)	0.0002	29 (25.00)	5 (13.89)	0.38 (0.11–1.23)	0.0999	111 (38.68)	8 (19.51)	0.23 (0.09–0.62)	0.0018	29 (45.31)	2 (25.00)
college	57 (49.14)	12 (46.15)	0.29 (0.11–0.78)	0.0115	63 (54.31)	20 (55.56)	0.69 (0.29–1.66)	0.1113	137 (47.74)	21 (51.22)	0.50 (0.23–1.10)	0.0813	26 (40.63)	5 (62.50)
father														
primary (ref.)	22 (18.97)	11 (40.74)			24 (25.53)	4 (11.11)			56 (19.58)	9 (22.50)			22 (34.38)	1 (12.50)
secondary	52 (44.83)	2 (7.47)	0.08 (0.02–0.38)	0.0002	30 (31.91)	7 (19.44)	1.40 (0.37–5.35)	0.6218	119 (41.61)	9 (22.50)	0.47 (0.18–1.25)	0.1239	24 (37.50)	3 (37.50)
college	42 (36.21)	14 (51.85)	0.67 (0.26–1.71)	0.3982	40 (42.55)	25 (69.44)	3.75 (1.16–12.09)	0.0210	111 (38.81)	22 (55.00)	1.23 (0.53–2.86)	0.6241	18 (28.13)	4 (50.00)

a Due to the small sample size in the subgroups, analysis was not possible.

Among the 8344 participants, 671 (8.04%) reported exposure to e-cigarette promotion. Among those exposed to promotion, exposure patterns varied by channel. Shop promotions reached 168 respondents (25.04% of those exposed to promotion), including 142 standard e-cigarette users and 26 who reported psychoactive substance use. Internet promotions demonstrated the highest reach with 367 respondents (54.69% of those exposed to promotion), including 327 standard e-cigarette users and 40 who reported psychoactive substance use. Club or social venue promotions reached 166 respondents (24.74% of those exposed to promotion), including 130 standard e-cigarette users and 36 who reported psychoactive substance use, representing the highest proportion of substance users among all promotional channels. Other forms of promotion showed the lowest reach with 80 respondents (11.92% of those exposed to promotion), including 72 standard e-cigarette users and 8 who reported psychoactive substance use.

These findings indicate varying levels of association between different promotional channels and psychoactive substance use in e-cigarettes. While internet promotions reached the largest audience, club or social venue promotions demonstrated the strongest association with psychoactive substance use, followed by shop promotions. Other forms of promotion showed both the lowest reach and relatively moderate levels of association with psychoactive substance use. This information provides valuable insights into the differential impact of promotional strategies on high-risk behaviors among adolescent e-cigarette users. To further examine these relationships, detailed statistical analyses of promotion exposure patterns and their associations with psychoactive substance use across different demographic groups were conducted. The analysis revealed several statistically significant relationships. Exposure to promotions in urban areas with populations ≥500 000 inhabitants was consistently associated with higher rates of psychoactive substance use. Adolescents in large cities were more likely to use psychoactive substances when exposed to club promotions (OR = 7.33, 95% CI: 2.37–22.70, p = 0.0002) and internet promotions (OR = 5.22, 95% CI: 2.06–13.22, p = 0.0002) compared to rural counterparts.

Socioeconomic factors played a significant role. Adolescents with higher personal disposable income (>35 EUR monthly) exposed to promotions demonstrated higher rates of psychoactive substance use, particularly evident in internet promotions (OR = 2.73, 95% CI: 1.31–5.67, p = 0.0056).

Parental education showed varying effects. Maternal secondary education demonstrated a protective effect compared to primary education, particularly evident in shop promotions (OR = 0.09, 95% CI: 0.02–0.38, p = 0.0002) and internet promotions (OR = 0.23, 95% CI: 0.09–0.62, p = 0.0018). However, paternal college education was associated with increased substance use in club promotions (OR = 3.75, 95% CI: 1.16–12.09, p = 0.0210).

Gender and age group did not demonstrate statistically significant associations with psychoactive substance use in relation to promotion exposure across most channels. These findings underscore the complex interplay between sociodemographic factors, promotion exposure, and psychoactive substance use in e-cigarettes among Polish adolescents, highlighting the particular significance of urban environments, higher income levels, and varying effects of parental education as key moderating factors.

#### Association between influencer marketing and psychoactive substance use

The examination of e-cigarette influencer marketing exposure and its association with psychoactive substance use among Polish adolescents revealed distinct patterns across various channels (Table 4). Four primary influencer marketing channels were investigated: shop-based activities, club or social venue promotions, internet marketing, and other influencer-driven promotions.

**Table 4.** Association between e-cigarette influencer marketing exposure and psychoactive substance use among Polish teenagers aged ≥15 years (N = 8344) – a cross-sectional study in Poland, 2019–2020

Variable	Influencer													
	shop				club				internet				other	
	psychoactive substances use [n (%)]		OR (95% CI)	p	psychoactive substances use [n (%)]		OR (95% CI)	p	psychoactive substances use [n (%)]		OR (95% CI)	p	psychoactive substances use^a^ [n (%)]	
	no	yes			no	yes			no	yes			no	yes
Gender														
male (ref.)	70 (65.42)	18 (66.67)			56 (65.12)	20 (62.50)			286 (58.97)	35 (60.34)			37 (57.81)	9 (75.00)
female	37 (34.58)	9 (33.33)	0.95 (0.39–2.31)	0.9030	30 (34.88)	12 (37.50)	1.12 (0.48–2.60)	0.7919	199 (41.03)	23 (39.66)	0.94 (0.54–1.65)	0.8404	27 (42.19)	3 (25.00)
Age group														
15–17 years (ref.)	71 (66.36)	22 (81.48)			56 (65.12)	23 (71.88)			378 (77.94)	44 (75.86)			54 (84.38)	7 (58.33)
≥18 years	36 (33.64)	5 (18.52)	0.45 (0.16–1.28)	0.1275	30 (34.88)	9 (28.13)	0.73 (0.30–1.78)	0.4878	107 (22.06)	14 (24.14)	1.12 (0.59–2.13)	0.1115	10 (15.63)	5 (41.67)
School type														
grammar (ref.)	41 (38.32)	8 (29.63)			30 (34.88)	8 (28.13)			210 (43.30)	21 (37.93)			26 (40.63)	5 (41.67)
other	66 (61.68)	19 (70.37)	1.48 (0.59–3.68)	0.4022	56 (65.12)	19 (71.88)	1.27 (0.50–3.25)	0.6142	275 (56.70)	34 (62.07)	1.24 (0.70–2.19)	0.4673	38 (59.38)	7 (58.33)
Place of residence														
rural (ref.)	41 (38.32)	15 (55.56)			38 (44.19)	14 (43.75)			208 (42.89)	24 (41.38)			19 (29.69)	2 (16.67)
city														
<500 000 inhabitants	61 (57.01)	4 (14.81)	0.18 (0.06–0.58)	0.0019	41 (47.67)	6 (18.75)	0.40 (0.14–1.14)	0.0798	255 (52.58)	24 (41.38)	0.82 (0.46–1.49)	0.5014	38 (59.38)	7 (58.33)
≥500 000 inhabitants	5 (4.67)	8 (29.63)	4.37 (1.23–15.48)	0.0166	7 (8.14)	12 (37.5)	4.65 (1.52–14.20)	0.0050	22 (4.54)	10 (17.24)	3.94 (1.67–9.30)	0.0009	7 (10.94)	3 (25.00)
Monthly income														
≤35 EUR (ref.)	51 (47.66)	5 (18.52)	4.01 (1.42–11.37)	0.0061	38 (44.19)	7 (21.88)			275 (56.70)	20 (34.48)			34 (53.13)	4 (33.33)
>35 EUR	56 (52.34)	22 (81.48)			48 (55.81)	25 (78.13)	2.83 (1.10–7.24)	0.0265	210 (43.30)	38 (65.52)	2.49 (1.41–4.40)	0.0013	30 (46.88)	8 (66.67)
Education														
mother			0.03 (0.00–0.22)	<0.0001										
primary (ref.)	12 (11.21)	11 (42.31)	0.29 (0.11–0.79)	0128	12 (13.95)	7 (21.88)			73 (15.05)	13 (22.81)			5 (7.81)	1 (8.33)
secondary	42 (39.25)	1 (3.85)			24 (27.91)	4 (12.50)	0.29 (0.07–1.17)	0.0731	167 (34.43)	8 (14.04)	0.27 (0.11–0.68)	0.0032	26 (40.63	5 (41.67)
college	53 (49.53)	14 (53.85)			50 (58.14)	21 (65.63)	0.72 (0.25–2.08)	0.5435	245 (50.52)	36 (63.15)	0.83 (0.42–1.64)	0.5824	33 (51.56)	6 (50.00)
father			0.15 (0.03–0.76)	0.0109										
primary (ref.)	25 (23.36)	8 (29.63)	1.33 (0.50–3.53)	0.5688	16 (18.60)	8 (21.62)			109 (22.47)	8 (15.38)			14 (21.87)	0 (0.00)
secondary	42 (39.25)	2 (7.41)			27 (31.40)	3 (8.11)	0.22 (0.05–0.96)	0.0344	194 (40.00)	14 (26.92)	0.98 (0.40–2.42)	0.9706	18 (28.13)	7 (58.33)
college	40 (37.38)	17 (62.96)			43 (50.00)	26 (70.27)	1.21 (0.45–3.22)	0.7032	182 (37.53)	30 (57.56)	2.25 (0.99–5.08)	0.0470	32 (50.00)	5 (41.67)

a Due to the small sample size in the subgroups, analysis was not possible.

Among the 8344 participants, 871 (10.44%) reported exposure to e-cigarette influencer marketing. Among those exposed to influencer marketing, exposure patterns varied by channel. Shop-based activities reached 161 respondents (18.48% of those exposed to influencer marketing), including 134 standard e-cigarette users and 27 who reported psychoactive substance use. Internet-based strategies demonstrated the highest reach with 601 respondents (69.00% of those exposed to influencer marketing), including 543 standard e-cigarette users and 58 who reported psychoactive substance use. Club or social venue promotions reached 150 respondents (17.22% of those exposed to influencer marketing), including 118 standard e-cigarette users and 32 who reported psychoactive substance use, representing the highest proportion of substance users among all influencer marketing channels. Other forms of influencer marketing showed the lowest reach with 88 respondents (10.10% of those exposed to influencer marketing), including 76 standard e-cigarette users and 12 who reported psychoactive substance use. These findings indicate varying levels of association between different influencer marketing channels and psychoactive substance use in e-cigarettes. While internet-based strategies reached the largest audience, club or social venue promotions demonstrated the strongest association with psychoactive substance use, followed by shop-based activities. Other forms of influencer marketing showed both the lowest reach and relatively moderate levels of association with psychoactive substance use.

To further examine these relationships, detailed statistical analyses of influencer marketing exposure patterns and their associations with psychoactive substance use across different demographic groups were conducted. The analysis revealed several statistically significant relationships. Exposure to influencer marketing in urban areas with populations ≥500 000 inhabitants was consistently associated with higher rates of psychoactive substance use across multiple channels. Adolescents in large cities (≥500 000 inhabitants) were more likely to use psychoactive substances when exposed to shop-based activities (OR = 4.37, 95% CI: 1.23–15.48, p = 0.0166), club promotions (OR = 4.65, 95% CI: 1.52–14.20, p = 0.0050), and internet marketing (OR = 3.94, 95% CI: 1.67–9.30, p = 0.0009) compared to rural counterparts.

Socioeconomic factors played a significant role across all channels. Adolescents with higher personal disposable income (>35 EUR monthly) exposed to influencer marketing demonstrated higher rates of psychoactive substance use, particularly evident in shop-based activities (OR = 4.01, 95% CI: 1.42–11.37, p = 0.0061), club promotions (OR = 2.83, 95% CI: 1.10–7.24, p = 0.0265), and internet marketing (OR = 2.49, 95% CI: 1.41–4.40, p = 0.0013).

Parental education showed varying effects. Maternal secondary education demonstrated a protective effect compared to primary education, particularly evident in shopbased activities (OR = 0.03, 95% CI: 0.00–0.22, p < 0.0001) and internet marketing (OR = 0.27, 95% CI: 0.11–0.68, p = 0.0032) for maternal education, with similar trends for paternal education. However, paternal college education was associated with higher substance use in Internet marketing (OR = 2.25, 95% CI: 0.99–5.08, p = 0.0470). Gender and age group did not demonstrate statistically significant associations with psychoactive substance use in relation to influencer marketing exposure across most channels.

These findings underscore the complex interplay between sociodemographic factors, influencer marketing exposure, and psychoactive substance use among Polish adolescents, highlighting urban environments, higher income levels, and varying effects of parental education as key moderating factors.

## DISCUSSION

This study demonstrates significant associations between multi-channel e-cigarette marketing exposure and psychoactive substance use among Polish adolescents. Analysis revealed distinct patterns of exposure across different marketing channels, with substantial variations based on urban environments, socioeconomic factors, and parental education. The observed urban-rural disparity was particularly pronounced, with adolescents in large cities showing substantially higher odds of psychoactive substance use when exposed to e-cigarette marketing (OR = 3.94–7.33, p < 0.001). Socioeconomic analysis revealed that higher-income adolescents demonstrated increased likelihood of substance use when exposed to marketing (OR = 1.81–4.01, p < 0.01). The influence of parental education emerged as complex, with maternal secondary education associated with lower odds of substance use (OR = 0.23–0.38, p < 0.01) compared to primary education. Among various marketing channels, club or social venue promotions demonstrated the strongest association with psychoactive substance use. These findings elucidate the complex interactions between marketing exposure, demographic factors, and high-risk behaviors among adolescent e-cigarette users, contributing new insights to the existing literature on this critical public health issue.

The socioeconomic gradient observed in this study, wherein higher-income adolescents exhibited an increased likelihood of substance use when exposed to e-cigarette marketing, challenges some previous findings. While Hartwell et al. [25] associated lower socioeconomic status with higher e-cigarette use, this study's results suggest a more nuanced relationship when considering marketing exposure and psychoactive substance use. This aligns with Leventhal et al. [26], who identified socioeconomic status as a significant predictor of e-cigarette use initiation.

The authors' findings on parental education further elucidate this complexity. Anyanwu et al. [27] reported that higher parental education was associated with increased e-cigarette use among U.S. adolescents, partially corroborating this study's results showing higher odds of substance use among those with college-educated fathers exposed to certain marketing channels. However, a protective effect of maternal secondary education was observed, contrasting with Perikleous et al. [28], who found higher maternal education associated with increased e-cigarette use among Cypriot adolescents. This suggests that the role of parental education in moderating e-cigarette marketing effects may be more intricate than previously conceived, warranting further investigation.

Additionally, this study's study revealed varying impacts of different e-cigarette marketing channels on psychoactive substance use. Among club or social venue promotional channels, the highest likelihood of psychoactive substance use was observed for adolescents in large cities compared to rural areas. These findings extend the research of Mantey et al. [29] regarding differential effects of e-cigarette marketing across various media channels. The authors' findings on influencer marketing corroborate those of Vogel et al. [30], who demonstrated that exposure to e-cigarette content on social media, particularly from influencers, was associated with increased e-cigarette use among adolescents.

The authors' findings underscore the complex interplay between e-cigarette marketing, sociodemographic factors, and high-risk behaviors among adolescents, highlighting the need for nuanced, context-specific approaches to e-cigarette regulation and prevention strategies, particularly in urban areas and among higher-income youth.

This study has significant implications for public health strategies and policy development. The strong association between marketing exposure and psychoactive substance addition of e-liquids necessitates more stringent regulations on e-cigarette advertising, especially in urban areas and through digital platforms. Comprehensive bans on e-cigarette marketing across all channels should be considered, with emphasis on social media and influencer marketing that disproportionately target youth. Prevention efforts should be tailored to reach higher-income youth, challenging conventional focus on lower socioeconomic groups. The protective effect of maternal secondary education suggests potential benefits of targeted educational interventions for parents.

Enhanced surveillance and monitoring systems are needed to track evolving trends of e-liquid modifications among adolescents. A multifaceted approach involving education, regulation, and community engagement is essential to address the complex issue of high-risk e-cigarette use among adolescents effectively.

Several strengths and limitations of this study warrant discussion. The study's strengths include its large, nationally representative sample across all Polish voivodships, comprehensive assessment of multiple marketing channels and high response rate (92.4%). The examination of various psychoactive substances, rather than focusing solely on cannabis or nicotine, offers a broader perspective on high-risk e-cigarette use behaviors. However, certain limitations should be considered when interpreting these results. The cross-sectional design precludes establishing causal relationships between marketing exposure and psychoactive substance use. Self-reported data may be subject to recall bias and social desirability bias, particularly regarding substance use behaviors. Additionally, while this study's operational definitions of marketing and promotion exposure were carefully constructed, the complex and often overlapping nature of these exposures in real-world settings may have led to some misclassification. These limitations highlight several important directions for future research.

## CONCLUSIONS

This study identifies demographic and environmental factors associated with illegal e-liquid modifications among Polish adolescents. While marketing exposure correlates with psychoactive substance use in e-cigarettes, this relationship likely reflects broader patterns of risk-taking behaviors rather than direct causation. The findings highlight particular vulnerability patterns, with pronounced effects in urban areas and through digital platforms, higher susceptibility among higher-income youth, and protective influence of maternal secondary education.

Future research should prioritize longitudinal studies to evaluate long-term health implications of e-cigarette modifications and understand the complex pathways leading to these behaviors. Investigating the efficacy of prevention strategies, elucidating underlying decision-making mechanisms, and evaluating the effectiveness of targeted interventions would yield valuable insights for future prevention efforts. Ongoing surveillance and adaptive policy responses are essential to address this evolving public health challenge effectively.
